# Observations on Venous Inflammation; with the Details of a Fatal Case of Phlebitis, Occurring after Venesection

**Published:** 1829-03

**Authors:** Henry S. Chinnock

**Affiliations:** Member of the Royal College of Surgeons, and Consulting Surgeon-Accoucheur to the Westminster Lying-in Hospital.


THE LONDON
### Medical and Physical Journal.

No. 361, VOL. LXI.] MARCH, 1829. [No. 33, *New Series.*

For many fortunate discoveries in medicine, and for the detection of numerous errors, the world is indebted to the rapid circulation of Monthly Journals; and there never existed any work, to which the Faculty, in Europe and America, were under deeper obligations than to the *Medical and Physical Journal of London*, now forming a long, but an invaluable series.—RUSH.

### ORIGINAL PAPERS, AND CASES,
### OBTAINED FROM PUBLIC INSTITUTIONS AND OTHER AUTHENTIC SOURCES.

### PHLEBITIS.

*Observations on Venous Inflammation; with the Details of a Fatal Case of Phlebitis, occurring after Venesection.* By HENRY S. CHINNOCK, Member of the Royal College of Surgeons, and Consulting Surgeon-Accoucheur to the Westminster Lying-in Hospital.

FEELING it to be the duty of every surgeon, at this particular moment, to submit all the information he possesses on the subject of inflamed veins to the notice of the profession, I have drawn up the following particulars of an interesting case that fell under my notice some time since; together with a few remarks.

The attention of the profession cannot have been otherwise than excited to an intense degree, by the highly interesting paper written on this subject by Mr. ARNOTT, which has lately been read before the Medical and Chirurgical Society, as well as by the valuable remarks made by the members during the discussions consequent on its introduction.

It is unnecessary, I conceive, to enter into a detail of Mr. Arnott's opinions, and the extent to which he carries his views on this subject, further than in the application of this case to his theory; inasmuch as every one of your readers have had equal opportunities with myself of collecting them from the report of the proceedings of the

Society of that night, as published in this Journal for the month of December last.

The case I refer to is that of a labouring man, of a full, robust, plethoric habit, between thirty and forty years of age. He had been bled, he stated, two or three days previously, at St. George's Hospital, in consequence of a fall from a ladder, but had not been prevented from attending to his ordinary occupation during the few days subsequent to the operation.

On my first interview with him, he did not complain of any urgent symptom; neither did I observe the slightest reason for further medical treatment than the simple exhibition of a calomel and saline purge. His principal complaint was a feeling of heaviness about the head, with the very general expression that he "felt ill," excessively "weak and stiff," and some sense of chilliness; sensations he conceived to be wholly proceeding from the accident above referred to.

The same general complaint was made, and the same indefinite set of symptoms continued, for the two succeeding days, with the addition only, on the evening of the second, of more than ordinary heat and dryness of skin, some additional sharpness and activity in the pulse, an appearance of restlessness and irritability, together with a more decided sense of chilliness, &c.

Although this state of things made me more anxious in my examinations, and the character and the peculiar sharpness of the pulse induced me to suspect the existence of inflammatory action, I could not obtain from my patient's account any further clue to the mischief, or any more satisfactory statement than on any of the previous days. He felt no local pain; neither did he on this occasion complain of any urgent or distressing symptom.

It appearing to me to be a genuine case of synocha, I bled him largely; ordered a calomel and saline purgative, with tartarized antimony, to be again exhibited; and the fever diet to be rigidly adhered to.

I found my patient on the next morning in a most excited condition; the feverish symptoms were much higher, the skin was excessively hot and dry, the countenance flushed, tongue coated with a thick white fur, pulse hard and full. He complained of severe headach; his respiration was somewhat oppressed and painful, but the sensorium was little affected.

I still considered it a case of common inflammatory fever, unaccompanied by any visceral or cerebral affection; inasmuch as, in answer to my most minute inquiries and examinations, there was no additional complaint or pain; no further local affection than on any of the previous days, only a general aggravation of the same set of symptoms. I immediately determined on the further abstraction of blood; and, on proceeding to perform the operation, I attempted to lift and examine the arm he had been, six days previously, bled in at the hospital, prior to the usual application of the bandage, (I bled him in the left arm on the day before,) when there was an involuntary shudder on the part of the poor fellow, accompanied with an expressed wish that I would again open a vein in the left arm; as, ever since he was bled at the hospital, the right had felt "stiff and uncomfortable," and within the last two days so tender that he could scarcely move it, or bear it touched, without considerable pain and inconvenience.

I naturally inquired why he had not previously mentioned it, especially as I had so particularly examined him for local symptoms or pain. He excused himself by observing, that he considered it was usual that the arm should feel painful and uncomfortable subsequent to venesection, and with that impression had moved it but little; he consequently felt it only comparatively inconvenient, and should not now have mentioned it but for the sudden and acute "twinge" produced on my disturbing it.

On examining the arm, I found the median cephalic was the vein that had been opened. The orifice had apparently partially united by the first intention, but the wound was now disposed to separate, with everted edges; there was a blush of erysipelatous inflammation, and considerable puffiness, surrounding the orifice, and an irritable condition of the wound itself. There was, as well, much general tumefaction of the arm and shoulder, extending round the axillary region. It had an œdematous feeling to the touch, but left no pitting. He complained of a sense of "stiffness" and "soreness," similar to that experienced in erysipelas; the pain on pressing it was exquisitely acute.

My attention was not particularly directed to the course of the vein, or any pathognomonic symptom of phlebitis, on this occasion; neither do I conceive I could have obtained any satisfactory diagnostic while there was so much general mischief, and such an extent of tumefaction. I, without further delay or examination, bled him again in the left arm, ordered twenty leeches to be instantly applied to the part, a fomentation of poppies to be freely used, and the limb to be completely enveloped in a linseed poultice. Saline and antimonial medicines were prescribed, with a calomel and opium pill at night, to be followed by a brisk aperient on the succeeding morning.

On my visit next day, I found he was much less heated: he had slept tolerably well in the course of the night, and expressed himself much relieved of the local symptoms by the bleeding, leeches, and fomentations, and generally stated himself more composed and comfortable. His tongue was equally loaded as yesterday, and the coating of a darker colour, although the bowels had been freely and very satisfactorily relieved, with this exception, that the feces discharged were not natural; they were dark and fetid. Urine very high coloured. The skin was still dry, but not so hot. His countenance was anxious, and he appeared generally irritable; his intellectual faculties were more disturbed; the pulse was 120, sharp and small. He complained of a painful throbbing sensation in the axilla, and a stronger sense of tension around the shoulder and the boundaries of the pectoral muscles; and, on examination, there was, in the whole neighbourhood of the armpit, evidently increased tumefaction and sensibility. He had complained of chilliness about three hours previously, amounting almost to a complete rigor. I ordered twelve more leeches to be applied, the poultice and fomentation to be continued, and a mixture composed of camphor julep, tincture of henbane, and acetate of ammonia, to be exhibited; and the opiate at bedtime to be repeated.

There was something in the character of the pulse observable on this visit, that indicated the necessity of still further depletion; but the anxious appearance of the countenance, the state of the tongue, skin, and sensorium, giving a typhoid character to the type of fever, and there being evident signs of suppuration in the axilla, I was induced to defer the adoption of very active measures.

On the next (the fifth) morning, there were more evident symptoms of distress: he had passed a restless, indeed, as he stated, a "miserable" night; the leeches had produced but very trifling relief. The only comfort he experienced was in the use of the fomentation. There was a much greater degree of irritability than on any previous occasion. He had two distinct shivering fits in the night, and occasional perspiration. The arm was more painful and distressing than ever; the pain on the shoulder and armpit he described as agonizing. There was still considerable general tumefaction; the axillary region, especially, was much more enlarged than yesterday. The swelling was of a peculiar nature: it was very diffuse, very extensive; it had no particular prominence or depending part, but bore a general tympanitic character. On pressing it, it did not pit; it gave a feeling of elasticity. There was a sense of fluctuation, a sort of undulating character, that indicated the existence of fluid. After a very careful examination, being convinced of the presence of inflammatory secretion, I made a free incision with a common sharp-pointed bistoury, which gave exit to a great quantity of healthy pus. Great comfort and relief was instantly experienced; the tension and pain was immediately relieved; and, soon after the application of a poultice, he fell into a sweet composed sleep.—Some beef-tea, with arrowroot, farinaceous puddings, &c. were allowed, and the same medicines continued.

My attendance was requested very early on the succeeding day, when I found my patient much worse. He awoke from the sleep relieved, but had gradually been getting worse. There was much additional excitement; the pulse was full, sharp, and rapid; the tongue white, the countenance flushed; and there was every symptom present of fresh inflammatory action. On examining the arm, I found the discharge still continuing from the axilla; tumefaction very trifling. He complained of acute "shooting pains"

running down the arm, from the shoulder to the forearm. On examining it, I found the limb much smaller: that is to say, the morbid puffiness and tumefaction much less; the whole course of the cephalic vein was marked with a reddened line of inflammation, and so hardened as to be distinctly felt like a cord under the skin, and almost perceptibly forming the line of distinction between the clavicular portion of the deltoid and pectoralis major muscles. Great pain was experienced on pressure in the course of the vein; there was a slight weeping of pus from the orifice of the vein, increased by pressing downwards. The pain was now confined to the course of the vein; he could move his arm more easily than before, and was enabled to turn in bed, and breathe with less suffering and inconvenience. His spirits began to sink; he expressed himself worn out by the disease and remedies, and seemed desirous that nature should have her course, rather than submit to further medical treatment. He was, however, induced to submit to the application of eighteen leeches to the course of the vein, and the continuance of the fomentation and poultice. Saline medicines, with henbane and opiates, composed the principal items in the prescriptions. Whey, thin arrowroot, &c. were directed as his principal articles of diet; and perfect quiet was strictly enjoined.

In the evening of this day I again saw him: the leeches had produced great relief in the local symptoms; the line of inflammation was much less distinct; the vein was not so hard, neither was there any thing like the degree of sensibility complained of in the morning; the pulse was very rapid and *sharp*; his intellect was much disturbed; he appeared very anxious and irritable, and not at all disposed to sleep.—I ordered a full dose of opium, and directed the poultice and fomentation to be continued.

The next (the seventh) day, typhoid symptoms were very evident. He had been excessively restless during the night, occasionally wandering in his imagination, but not decidedly delirious. The skin was very dry, the mouth parched, the countenance excessively anxious, urine very scanty, pulse very rapid, and breathing hurried. The integuments in the axilla were very loose and flabby, had a livid appearance, especially in the neighbourhood of the opening. A discharge of thin pus, of a sanious character, with a fetid odour, was exuding from it, and a secretion of the same description weeping from the orifice in the bend of the arm. The red line of inflammation had completely disappeared, the vein was much softer, and its course scarcely perceptible. He again complained of oppression in the chest, in contradistinction to that of painful respiration before complained of. He had a sense of fulness in the right side, and a difficulty in "drawing over a breath:" when he wanted to inspire, he was obliged, he said, to "stop short half way," otherwise he feared he should be "choked." It produced no pain, as it did three or four days since. He had no cough, but constantly felt a choking sensation,

He was unable to lie completely in a horizontal position, and was consequently raised to a semierect posture. He now directed my attention to the opposite axilla, where he had sensibly felt pulsation and a sense of "fulness and stiffness," similar to that at first occurring in the right. There was a slight feeling and appearance of tumefaction, with some tenderness and perceptible increase of heat.

Poultices and fomentations were still resorted to as the only means of relief for the local affection. Ammonia, aromatic confection, and opium, were the medicines. For diet, he was ordered every description of light nutritious broth, with wine.

He rapidly sank from this day. The typhoid symptoms became more urgent; he passed miserable nights; was at last delirious; his skin became as dry as parchment, and his tongue like a nutmeg-grater; he had subsultus tendinum; the pulse was fluttering, and scarcely to be counted. The wound of the arm and axilla assumed a completely livid aspect; the discharge was much diminished and ichorous. The left axilla was during this time a great source of annoyance to him; the tumefaction had increased, there was evident fluctuation; but he would not submit to the use of the lancet; and on the last day of his life the integuments assumed the same disposition to gangrene as in the opposite limb. He during this time drank his wine and porter (which he was of course liberally allowed,) greedily, and resorted to his stimulating medicines with great avidity; and at length died in a complete state of exhaustion, on the evening of the ninth day of my attendance.

On the succeeding morning I examined the body. The arm first attracted my attention. The orifice opened distinctly into the vein. On introducing air through a blowpipe, I found the vein completely pervious. There was much induration, thickening, and adhesion of the integuments with the subjacent fascia and surrounding parts, so that it was only with considerable care and difficulty I was enabled to examine the whole course of the vein. The median-cephalic and cephalic veins were double their natural size; the coats of the vein were considerably thickened; the external tunic, where it was distinctly detached from the neighbouring parts, was much redder and more vascular than natural, putting on a completely arborescent appearance, produced (I should conceive) by an injected state and increased size of the vasa vasorum. There was not the slightest appearance of blood in the cavity of the vessel: it was partially filled with purulent matter, in patches like clotted cream; its inner coat was very red, and much thickened; its general appearance was that of an artery rather than a vein. On tracing it towards its termination in the axillary vein, the same appearances were observed, till within about half an inch of its entrance into the principal trunk. The median-basilic and the basilic as well showed feeble traces of inflammatory action. There was in the interstices of the deltoid, pectoralis major and minor, coraco-brachialis, and brachialis internus muscles, a deposition of dark-coloured semifluid matter. The fibres of the pectoralis major, subscapularis, and other muscles, constituting the boundaries of the axillary space, were gangrenous. The whole of the adjoining cellular tissue was in a sloughing condition, and injected with, or rather floating in, matter of an ichorous and sanious character. There was no trace of disease further than an inch below the orifice, excepting an injected state and tumefied condition of the cellular tissue; the veins were perfectly healthy, and not adherent to the neighbouring parts.

I found in the left axilla from an ounce to an ounce and a half of thin grumous fluid. The surrounding parts were perfectly healthy, except those immediately forming the boundaries of the sac, which were almost in a sloughing condition.

I pursued my examination to the chest, to account for the minor degree of oppression complained of by the patient just prior to death, as well as the pain and difficulty of respiration in the commencement of the disease;* when, much to my surprise, I found from four to six ounces of semipurulent matter floting in the right cavity of the chest. There was very trifling adhesion of the pleura of the right side, but no further trace of inflammatory action. The right lung was in a complete state of *engorgement;* on cutting into it, a yellow mucilaginous fluid first escaped, and subsequently dark grumous blood. The structure of the lung was not materially altered. There was not the slightest vestige of disease in the opposite side of the chest; no more fluid than natural. The heart and pericardium healthy.

The liver, brain, and abdominal viscera were not examined.

I have been thus particular in the details of this case, as far as my memory, assisted with the few notes I took of the prominent features of it, would allow me, inasmuch as I think it one of a most interesting character to the profession. It was, without exception, the most active, the most alarming instance of this species of disease I ever met with. The case was evidently one of simple excitement, simple inflammatory fever, consequent on that general constitutional disturbance which usually attends any violent or accidental shock of the nervous or circulatory system. The local affection, I conceive, was not produced by a foul lancet, or the introduction of morbific matter, but was simple, common

* The painful and difficult respiration that distressed the man so much during the fourth and fifth days of my attendance, can, I think, be sufficiently accounted for by the inflammation extending to the cellular tissue, in connexion with, and thus implicating the action of the pectoralis major, minor, serratus anticus, and other muscles connected to the ribs; there being no signs whatever during life, nor (as will be seen by the account of the dissection) after death, of the existence of inflammation within the chest. The state of oppression felt on the two last days of his life was evidently the effect of purulent deposition.

inflammation, occurring in a habit predisposed to diseased action, such as is constantly happening from wounds or injuries of any part or any tissue, in a deranged or diseased constitution, or during febrile excitement. The cellular tissue of the arm was the part first inflamed: the vein was most certainly not, I think, at all implicated in the mischief at the first development of the local symptoms; neither was it, I conceive, in the least affected till the inflammation had nearly run its course, indeed not till the commencement of suppuration, till the actual secretion of pus. Very soon after the first rigor; and almost immediately after the first exudation of inflammatory secretion from the orifice, we had symptoms of reaction. On the approach of fresh inflammation, very shortly, indeed, after these symptoms, my attention was directed to the vein: then it was I observed its hardened condition, the evident red streak of inflammation, as I have before remarked as forming the line between the two muscles; then it was the whole train of symptoms accompanying phlebitis made their appearance in succession, marking it as a genuine case of that disease. In addition to the local symptoms, the rapid change from a state of excitement to that of exhaustion: in short, the almost instant approach of typhoid fever, the deposition of matter in the opposite axilla, and the loaded, the oppressed condition of the lung.

Whether the inflammatory effusion from the subjacent tissue entered the orifice of the vein, and immediately mixing with the current of blood, was conveyed to the heart through the axillary, subclavian, and cava veins; whether it insinuated itself (if I may use the expression) into the tube, and thus excited inflammation of the internal tunic of that vessel, and the consequent effusion of lymph thus transmitted through the venous canal to the system; or whether, in accordance with Mr. Hunter's expressed opinion on this point, "that in all cases of violent inflammation of the cellular membrane, whether spontaneous or in consequence of accident, as in compound fracture, or surgical operation, as in the removal of an extremity, that the coats of the larger veins passing through the inflamed part become also considerably inflamed, and that their inner surfaces take on the adhesive, suppurative, and ulcerative inflammations."* That the vein thus became

* Mr. Hunter further observes "it to be so common a case, that he has hardly ever seen an instance of suppuration in any part furnished with large veins where these appearances are not evident after death." He also states "that he has constantly found them in the bodies of those who have died implicated in the general mischief are, I consider, the only queries as to the immediate cause or origin of the phlebitic inflammation in this case.

I do not consider much time need be occupied in reflection. There were evident symptoms during life of the existence of inflammatory action in the whole course of the vein, and every satisfactory proof of the pre-existence of inflammation in the internal tunic of that vessel at the post-mortem examination; and I think the most plausible rationale is, that such inflammation was produced by the insinuation of inflammatory secretion from the adjoining cellular membrane; that the effusion of lymph was the consequence, and an admixture of pus with the circulating fluid thus produced the distressing train of secondary putrescent symptoms, and the consequent loss of the patient.

There cannot, I conceive, be a single doubt that the amalgamation of pus with the blood was the immediate cause of the poor fellow's death; otherwise, I would ask how could purulent matter have found its way into the opposite axilla, where there was no evidence of prior disease, or purulent depôts formed in the lung and in the cavity of the chest. If the vein had not thus been unfortunately implicated in the diseased action in the progress of the cellular inflammation, I am fully convinced the man would have recovered: the disease had evidently yielded to remedies, the constitution had stamina to support the loss of pus, the discharge was at first perfectly healthy, in short, every favorable symptom we could reasonably expect was present, till the development of the phlebitic affection. The constitution had necessarily been subject to extensive depletion to relieve the cellular inflammation, which was of itself of the most virulent and the most active character that ever fell under my notice, and the system could consequently ill afford submission to that mode of treatment likely to stop the progress of this fresh attack. If it had made its appearance under almost any other circumstances, I certainly should have hoped to derive much benefit from early depletion; but, happening as it did, there did not appear to me to be the slightest grounds for a favorable prognosis. The remarkable state of sinking, and the rapid approach of putrescent symptoms and death, after the from amputations, compound fractures, and mortifications." (See "Observations on Inflammation of the Internal Coats of Veins," by John Hunter, Esq. F.R.S. Transactions of a Society for the Improvement of Medical and Chirurgical Knowledge, p. 19. 1793.)

active phlebitic inflammation, prove clearly to my mind that nothing can be possibly done to save life after pus is thus conveyed into the system; after the inflammation once terminates in effusion.

The result of this case enables me to bear Mr. Arnott out, and to strengthen his views on the pathology of this disease. It may be added to the ten cases he related to prove the truth of his proposition, that "there is no evidence of the inflammation of the vein extending to the heart," and further increase the value of his aphorism, "that there is no direct relation between the degree of danger and the extent of vein inflamed," inasmuch as there was not in this instance the slightest trace of inflammation beyond the junction of the cephalic with the axillary, or much less in the subclavian or cava veins: and most decidedly do I coincide with him, not only reasoning from this individual case, but drawing my deductions from other species of disease, in his general conclusion, "that abscesses and inflammations which take place in remote situations after injuries of the extremities, of the head, or after parturition, are dependent upon the existence of phlebitis in the part *originally* affected; that the diseased action does not consist in mere metastasis, or change of situation in absorbed matter, but that the secondary local affections derive their peculiar characters from a change induced in the blood, by its admixture with the pus or other inflammatory secretions from the vein." The great similarity in the constitutional disturbance, the febrile excitement, and the approach of the disease in this case, to the train of symptoms that occurred in two well-marked cases of inflamed cellular tissue, caused by the introduction of morbific matter, enables me, as well as Mr. Arnott, to assert that there is a most striking resemblance between the symptoms of inflamed veins and those attending wounds received during dissection, or the introduction of morbid poison by puncture.

In reference to phlebitis generally, I cannot avoid looking back and adopting the humoral pathology. In addition to the facts above stated, and Mr. Arnott's high opinion on this subject, I refer to the well-known circumstance of the blood of those patients who have died of phlebitis being so constantly observed, after death, to be almost in a fluid condition; a fact, by the by, I am not aware that Mr. A. has referred to. I do then consider we may indeed be very fully justified in applying the humoral doctrine to this species of disease.

Taking up this view of the question, I see no chance of saving the life of a patient suffering from phlebitis, but by *early* depletion. If the inflammatory action of the disease be not arrested *in limine*, and the purulent effusion prevented, there is little chance of a favorable issue; and as the vitiation of the fluids is a secondary process, and evidently the cause of the great prostration in the powers of life, our object must clearly be to stop the course of the inflammation to such a termination. To obtain this end, general and topical bloodletting must be resorted to as our sheet-anchor. Topical bleeding cannot possibly be carried to too great an extent: I would completely cover the course of the vein inflamed with leeches, but would not recommend the too free abstraction of blood by venesection. I conceive we ought to be very guarded, very cautious, in drawing it from the general system. It can seldom be borne, unless in the very earliest stages.

Where the venous is not complicated with extensive inflammation of adjoining cellular tissue, I think much good may be derived by the application of cold above the immediate seat of disease; and, where the limb will admit of it, I think Mr. Hunter's remedy is very plausible, that of compressing the vessel, to promote the deposition of coagulum, the adhesion of the sides of the vessel, and consequently prevent the passage of the depraved fluid to the heart.

*Michael's Place, Brompton.*

---

